# Synthesis, Properties, and Enantioseparation of Four-Ring Racemic Smectics

**DOI:** 10.3390/ma19091719

**Published:** 2026-04-23

**Authors:** Edyta Wojda, Monika Zając, Paweł Perkowski, Magdalena Urbańska

**Affiliations:** 1 Institute of Chemistry, Military University of Technology, ul. Sylwestra Kaliskiego 2, 00-908 Warsaw, Poland; edyta.wojda@student.wat.edu.pl (E.W.); monika.zajac@wat.edu.pl (M.Z.); 2 Institute of Applied Physics, Military University of Technology, ul. Sylwestra Kaliskiego 2, 00-908 Warsaw, Poland; pawel.perkowski@wat.edu.pl

**Keywords:** synthesis, smectics, racemates, chiral chromatography, resolution

## Abstract

**Highlights:**

- Two smectic racemates contain the SmA and SmC_A_ phases.
- These racemates are good admixtures for the antiferroelectric mixtures.
- The synthesized racemates have a different mesomorphic situation than the analogous enantiomers.
- It is possible to separate the racemates into enantiomers using chiral chromatography.

**Abstract:**

The synthesis and phase behavior of two liquid crystalline racemates containing four aromatic rings, differing in the number of methylene groups, were reported. These materials form smectic phases, as was confirmed by dielectric spectroscopy. The mesomorphic properties of the studied racemates were compared with those of the appropriate (S) enantiomers previously synthesized. Since these materials are racemic mixtures, they were subjected to chiral separation by high-performance liquid chromatography. This research was conducted on two chiral columns based on polysaccharides. We identified optimal conditions that enable the baseline separation of these racemates, which can be scaled up for preparative purposes. Then, there is no need for repeated synthesis of chiral equivalents.

## 1. Introduction

**Figure 1:**
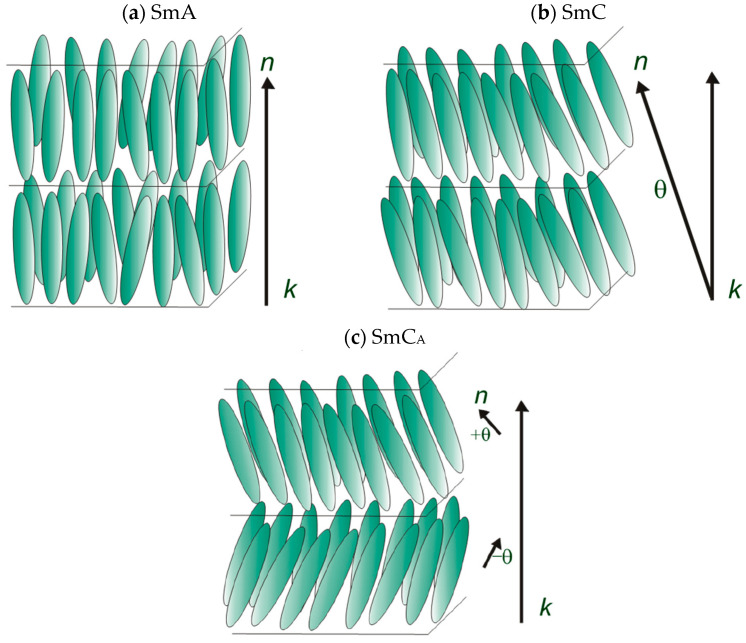
The structure of the three smectic phases.

The liquid crystal state has been known for over 130 years [1,2]. Liquid crystals are an intermediate phase between the liquid and crystalline states of matter, characterized by the ability to flow, a property characteristic of liquids, and, at the same time, by long-range molecular ordering, similar to that found in crystals. The anisotropic properties of liquid crystals have been leveraged in numerous applications, ranging from LCDs to stimuli-responsive polymers. A liquid crystal phase is thermotropic if its order parameter depends on temperature. At high temperatures, liquid crystals become isotropic liquids; at low temperatures, they become glassy or form molecular crystals. Most thermotropic liquid crystals are composed of rod-like molecules and admit nematic and smectic phases [3]. Liquid crystals belonging to the smectic class have a more ordered structure than those of the nematic class. In addition to orientational order, they exhibit positional order, leading to the formation of molecular layers. Smectic liquid crystals include compounds with only a smectic phase or those that form a smectic phase (at lower temperatures) and a nematic phase (at higher temperatures). The feature that unites all smectics is the layered structure. Still, they differ in the arrangement of molecules in the layer, the range and type of intermolecular interactions, and the range of positional and orientational order. Smectic phases are divided into orthogonal and tilted ones [2,4,5]. Smectic phases A, C, and C_A_ occur in materials that are presented in this work. In the smectic A phase (SmA), the director n and the optical axis are perpendicular to the smectic layer and parallel to the layer normal k (see Figure 1a). Smectic C phase—synclinic phase (SmC)—is a subphase of the oblique phases. It resembles the SmA phase, but the director in this phase is tilted at a specific angle (θ) relative to the layer, as shown in Figure 1b. This phase has two optical axes of the system. The SmC phase can be low-angle, with a tilt angle of 25–30°, or high-angle, with a tilt angle exceeding 40°. An important subphase of the tilted phases is the smectic C_A_ phase—anticlinic phase (SmC_A_), which is characterized by two adjacent layers (two-layer unit), in which the director n makes an angle +θ or −θ, with the direction defined by k, as shown in Figure 1c. We are exploring new materials with smectic phases as components of liquid crystalline mixtures [6,7,8,9,10,11,12]. The racemic mixtures discussed in this paper can be used as dopants in antiferroelectric mixtures [13,14]. Additionally, they can be separated into enantiomers using high-performance liquid chromatography [15,16,17,18,19,20]. Finding optimal separation conditions would avoid the costly synthesis of the corresponding enantiomers.

As part of our ongoing research on four-ring smectics, we synthesized two liquid crystalline racemates. We characterized their mesomorphic properties and tested whether they could be separated into enantiomers by chiral liquid chromatography (HPLC). Having previously separated three-ring esters, we investigated how the additional benzene ring in the structure affects enantioseparation. The results are presented in this work.

## 2. Materials and Methods

**Figure 2:**
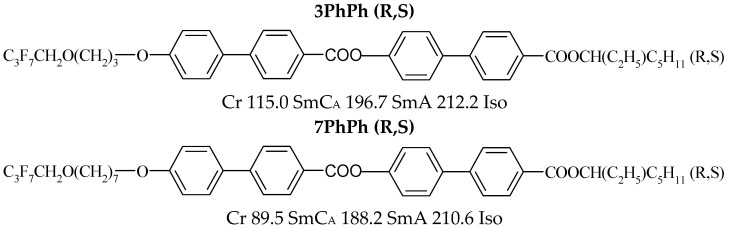
The structures, acronyms, and phase transition temperatures of smectic racemates.

The chemical structures for the designed and synthesized racemates are presented in Figure 2. The racemates have four benzene rings, differ in the number of methylene groups (r = 3 or 7), and have an achiral part synthesized based on (R,S)-3-octanol.

**Figure 3:**
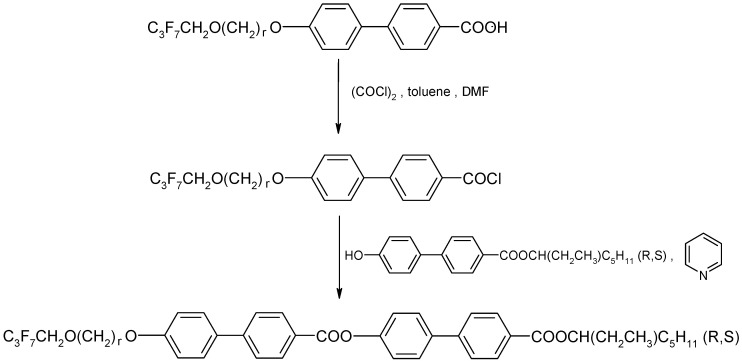
Synthetic route of racemates (r = 3 and 7).

The racemates were prepared by the same classical method, treating the chiral phenol with a biphenyl acid chloride in the presence of pyridine (see Figure 3). The synthesis was described in ref. [21] using the (S) enantiomers as an example. About 1 g of each racemic mixture was obtained.

The final products were purified by column chromatography in the first step and by crystallization from ethanol and acetone in the second step. The purity of these products was checked using a Shimadzu Prominence chromatograph equipped with an SPD-M20A diode-array detector (Shimadzu Co., Kyoto, Japan). The racemates have chemical purities greater than 99%. The optical purity of the (S) enantiomers exceeds 99%. The MS data for the racemates are presented in the Supplementary Materials (Figures S1 and S2). As a result of the ionization process of the racemates, the strong molecular ion with a captured sodium atom [M + Na]^+^ was observed for the studied racemates. For the racemate 3PhPh (R,S) MS: 785[M + Na]^+^ and for the racemate 7PhPh MS: 841[M + Na]^+^. The reaction yield was about 50% in all cases.

Proton (^1^H) and carbon-13 (^13^C) nuclear magnetic resonance (NMR) spectra in CDCl_3_ were collected on a Bruker Avance III spectrometer at room temperature. A comparison of NMR spectra confirmed that the real structures matched the planned structures.

For the racemate **3PhPh (R,S)**:

^1^H NMR (500 MHz, CDCl_3_) δ ppm 0.9 (t, 3 H) 1.0 (t, *J* = 7.3 Hz, 3 H) 1.3 (m, 4 H) 1.4 (m, 2 H) 1.7 (m, 4 H) 2.1 (dt, *J* = 12.2, 6.1 Hz, 2 H) 3.8 (t, *J* = 6.1 Hz, 2 H) 4.0 (t, *J* = 13.7 Hz, 2 H) 4.1 (t, *J* = 6.1 Hz, 2 H) 5.1 (m, 1 H) 7.0 (d, *J* = 8.9 Hz, 2 H) 7.3 (d, 2 H) 7.6 (d, 2 H) 7.7 (d, 3 H) 7.7 (d, *J* = 2.7 Hz, 3 H) 8.1 (d, 2 H) 8.3 (d, 2 H)
^13^C NMR (126 MHz, CDCl_3_) δ ppm 9.7; 14.0; 22.5; 25.1; 27.1; 29.5; 31.8; 33.7; 64.3; 67.8; 69.5; 76.3; 115.0; 122.2; 126.7; 127.0; 127.4; 128.4; 128.5; 129.7; 130.1; 130.8; 132.3; 137.9; 144.6; 146.0; 151.1; 159.3; 165.1; 166.2

For the racemate **7PhPh (R,S)**:

^1^H NMR (500 MHz, CDCl_3_) δ ppm 0.9 (t, 3 H) 1.0 (t, *J* = 7.5 Hz, 3 H) 1.3 (m, 4 H) 1.4 (m, 6 H) 1.5 (m, 2 H) 1.6 (m, 3 H) 1.7 (m, 3 H) 1.8 (m, 2 H) 3.6 (t, *J* = 6.4 Hz, 2 H) 3.9 (t, *J* = 14.0 Hz, 2 H) 4.0 (t, *J* = 6.6 Hz, 2 H) 5.1 (m, 1 H) 7.0 (d, 2 H) 7.3 (d, 2 H) 7.6 (d, 2 H) 7.7 (m, 6 H) 8.1 (d, 2 H) 8.2 (d, 2 H)
^13^C NMR (126 MHz, CDCl_3_) δ ppm 9.7; 14.0; 22.5; 25.0; 25.7; 26.0; 27.1; 29.1; 29.2; 29.4; 31.8; 33.7; 68.0; 73.2; 76.3; 115.0; 122.2; 126.6; 127.0; 127.3; 128.4; 130.1; 130.8; 132.0; 137.9; 144.6; 146.1; 151.1; 159.6; 165.1; 166.2

^1^H NMR and ^13^C NMR spectra of the synthesized smectics are presented in the Supplementary Materials (Figures S3–S6).

The phase behavior of the racemates was studied using differential scanning calorimetry (DSC) with a 204 F1 Phoenix microcalorimeter (NETZSCH-Gerätebau GmbH, Selb, Germany) at a heating/cooling rate of 2 °C·min^−1^. Optical textures were used to identify the liquid–crystalline phases, which were observed with an Olympus BX51 optical microscope (Shinjuku, Tokyo, Japan) equipped with a Linkam THMS-600 heating stage controlled by a TMS-93 temperature programmer (Linkam Scientific Instruments Ltd., Tadworth, UK).

Since it was difficult to identify all phases based solely on textures, additional dielectric measurements were performed. Dielectric spectroscopy is a useful method for identifying phases that appear in investigated racemates as temperature changes. Our laboratory has an impedance analyzer by Hewlett-Packard: the HP 4192A (Hewlett-Packard, Ltd., Tokyo, Japan). We used the measuring range from 100 Hz to 10 MHz. For measurements, we used self-made cells with gold electrodes to minimize the well-known high-frequency parasitic effects [22]. Such cells can be used for frequencies up to 5 MHz. The thickness of the used cells was approximately 5 µm, and the cells’ alignment was planar. We used polyimide SE130 (Nissan Chemical, Tokyo, Japan) as an aligning layer. Liquid crystals were heated and put in a measuring cell in the isotropic phase using capillary action. We conducted four measurement cycles to thoroughly characterize the material’s dielectric properties. All measurements were performed on the cooling cycles. We measured both without and with a 5 V DC field for the racemic mixtures.

Smectic racemates commonly exhibit the same phases as pure enantiomers [23,24,25]. The difference is that one does not observe helical structure and spontaneous polarization in racemic SmC_A_ and SmC phases. As a result, some of the dielectric modes observed in the enantiomer are absent in the racemate. The best example is the $P_{H}$-mode. It exists in an enantiomeric SmC_A_* phase, while it does not exist in its racemic form (SmC_A_). In enantiomer (SmC_A_*), three modes are usually observed: collective low-frequency $P_{L}$-mode, high frequency $P_{H}$-mode, and molecular S-mode (rotation around the short molecular axis). All three modes are Arrhenius-type—their relaxation frequencies depend on temperature (decreasing with decreasing temperature) [26,27,28,29]. Additionally, all three modes are modified by a DC field [30,31,32,33]. In racemate $P_{H}$- the mode vanishes, while $P_{L}$- and S-modes are still in electric response. Moreover, they are still enhanced by a DC field, as in the case of the enantiomer. Results obtained during dielectric measurements are presented in the Supplementary Materials in Figures S7–S13.

When the racemates presented in this paper were synthesized, we decided to compare their phase sequences with those of earlier-synthesized enantiomeric compounds. The enantiomers and their selected properties were previously presented in [21]. However, they have not been examined by dielectric spectroscopy. Both enantiomers 3PhPh and 7PhPh exhibit rich polymorphism of smectic phases: SmA*, SmC*, and SmC_A_*. This means we could expect SmA, SmC, and SmC_A_ phases in the synthesized racemates.

The Shimadzu LC-20AP HPLC system (Shimadzu Co., Kyoto, Japan), comprising a binary solvent delivery pump, an autosampler (SIL-10AP), a communications bus module (CBM-20A), a diode-array detector (SPD-M20A), and a fraction collector (FRC-10A), was utilized for the separation and detection of analytes. Data acquisition was performed by Shimadzu software (LabSolutions, 2010-2017, Shimadzu Co.). The measurements were made at room temperature. The mobile phase flow rate was 0.3 or 1.0 mL/min. The detection wavelength was set to 254 nm.

The mobile phase consisted of acetonitrile (ACN)/water, acetonitrile/methanol, or acetonitrile/ethanol. Elution was performed in an isocratic mode. The sample concentrations were about 0.6 mg/mL. The samples were dissolved in acetone because the solubility of the studied mixtures in acetonitrile is low at room temperature. The columns used had dimensions of 250 mm × 4.6 mm, a particle size of 5 µm, a pore size of 1000 Å, and a surface area of 30 m^2^/g (Dr. Maisch GmbH, Ammerbuch-Entringen, Germany). The chemical structures and names of the two chiral selectors used are presented in Table 1.

## 3. Results

### 3.1. Mesomorphic Properties of Racemates

**Figure 4:**
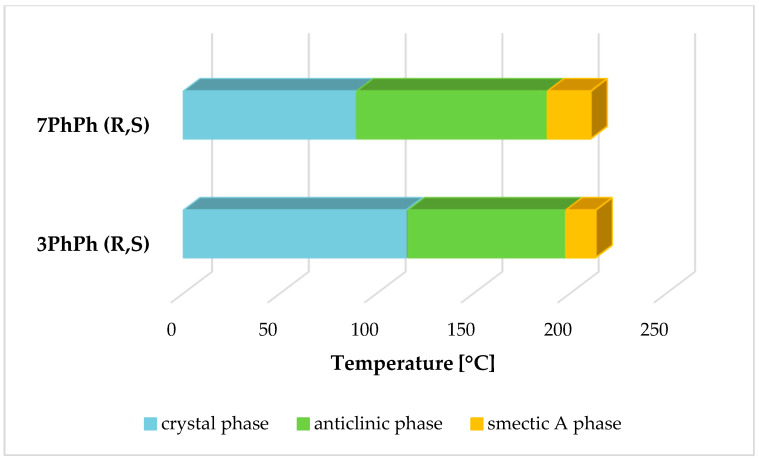
Phase transitions diagram for the racemates 3PhPh (R,S) and 7PhPh (R,S).

The transition temperatures and the associated enthalpy changes for the racemates are given in Table 2. The two racemates form the smectic A phase (SmA), which can be identified by characteristic textures observed with a polarized-light optical microscope. The remaining smectic phases were determined using dielectric measurements, as described above, because the textures were ambiguous, a common occurrence with racemates (see Figure 4). Dielectric investigations verify the phase sequence in the racemates 3PhPh (R,S) and 7PhPh (R,S). Both racemates exhibit the SmA and SmC_A_ phases, whereas, according to [21], both enantiomers exhibit the SmA*, SmC*, and SmC_A_* phases. Moreover, they confirm that a slight change in molecular structure leads to the disappearance of one mode ($P_{L}$-mode) in the electric response of the racemate 7PhPh (R,S).

The corresponding DSC curves for the racemates are included in the Supplementary Materials (Figures S14 and S15).

**Figure 5:**
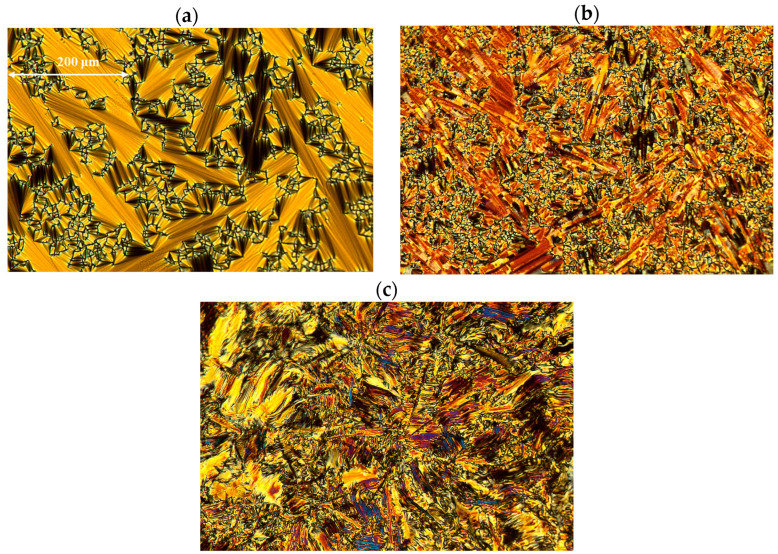
The photos of the textures obtained during the cooling cycle for the racemate 3PhPh (R,S) (**a**) in the SmA phase at 214.1 °C, (**b**) in the SmC_A_ phase at 192.2 °C, and (**c**) in the crystal phase at 68.9 °C.

**Figure 6:**
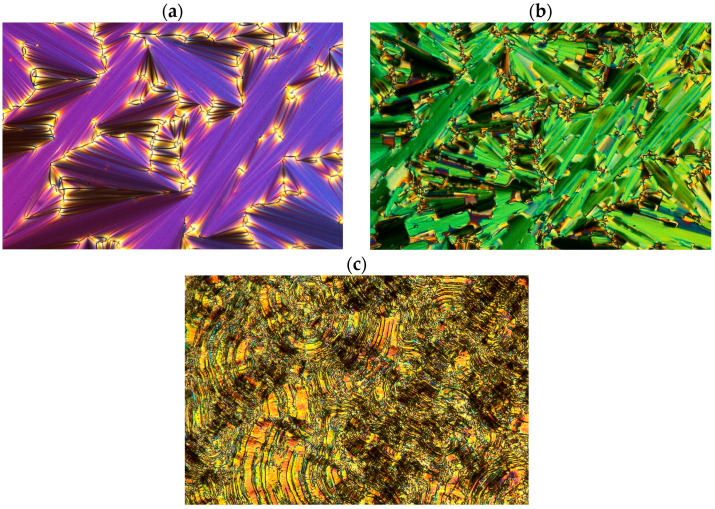
The photos of the textures obtained during the cooling cycle for the racemate 7PhPh (R,S) (**a**) in the SmA phase at 214.8 °C, (**b**) in the SmC_A_ phase at 192.2 °C, and (**c**) in the crystal phase at 93.4 °C.

Both racemates exhibit high clearing points (above 200 °C), which is attributed to the presence of four benzene rings in their structure. The racemate 3PhPh (R,S) has a higher melting point and melting enthalpy. Both racemates exhibit the anticlinic smectic phase (SmC_A_) in a very broad temperature range (above 80 °C). The racemate with seven methylene groups exhibits this phase in the range of about 100 °C. The SmA phase appears in a medium temperature range for these two racemates. The SmC_A_-SmA transition exhibits a small enthalpy value of about 1 kJ/mol. Characteristic textures (with a width of about 600 μm) for the observed phases are shown in Figure 5a–c for the racemate with r = 3 and in Figure 6a–c for the racemate with r = 7.

**Figure 7:**
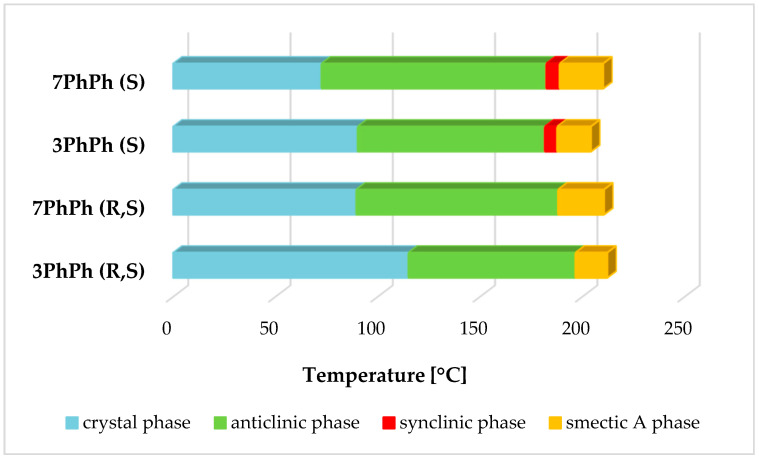
Phase transitions diagram for enantiomers and racemates. The (S) designation has been intentionally added to the enantiomer acronyms in the graph.

For the (S) enantiomers-3PhPh and 7PhPh [21], high clearing temperatures (above 200 °C) are also observed. The observed phase sequence for the enantiomers is Cr-SmC_A_*–SmC*–SmA*–Iso. Both enantiomers exhibit a very wide temperature range for the SmC_A_* phase. The SmC* phase occurs over a very narrow temperature range, while the SmA* phase appears in the intermediate temperature range. The enantiomer 3PhPh has the lower melting point of the two compounds. There is a significant difference between racemates and (S) enantiomers in their phase sequences and ranges of occurrence, as shown in Figure 7. In Figures S16 and S17, DSC curves for the enantiomers showing the occurrence of the SmC* phase have been added to the Supplementary Materials.

**Figure 8:**
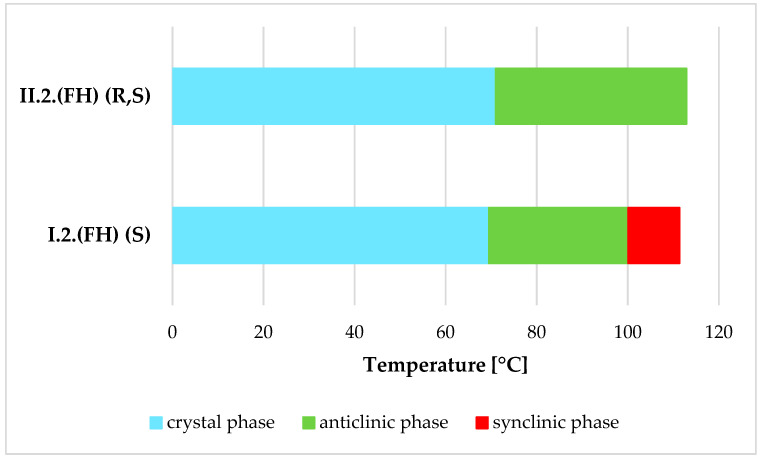
Phase transitions diagram for enantiomer and racemate with a phase situation similar to that of the studied materials.

Differences in the mesomorphic properties of racemates and enantiomers have been encountered before and involve the presence or absence of additional phases in the respective enantiomers or racemates. A situation similar to the one presented here occurs, for example, in a three-ring compound with a short oligomethylene spacer (r = 2) and a monofluorinated benzene ring, where the racemate exhibits only the SmC_A_ phase, whereas the enantiomer exhibits both the SmC_A_* and SmC* phases, as shown in Figure 8 [18].

### 3.2. Enantioseparation of Racemates

**Figure 9:**
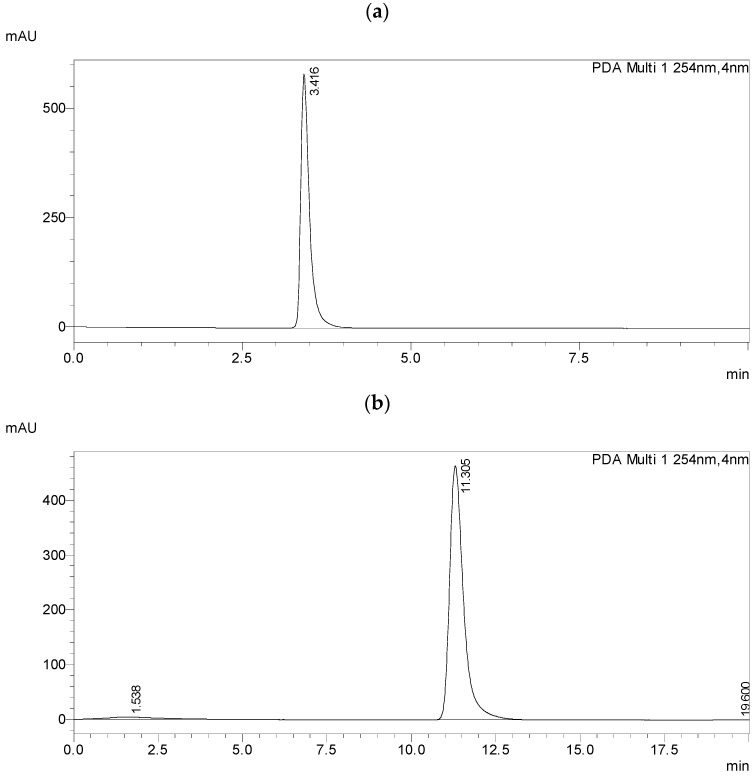
Chromatograms of (**a**) racemate 7PhPh (R,S) and (**b**) racemate 3PhPh (R,S) in the solvent system ACN/WATER in the ratio 99:1 on the ReproSil Chiral-MIG column, flow rate (**a**) 1 mL/min, (**b**) 0.3 mL/min.

On the ReproSil Chiral-MIG column, none of the racemic mixtures separated in the acetonitrile/water system (99:1, *v*/*v*), as shown in Figure 9a for the mixture 7PhPh (R,S); even reducing the flow rate did not help, as shown for the mixture 3PhPh (R,S) in Figure 9b. The analysis time was increased almost fourfold.

**Figure 10:**
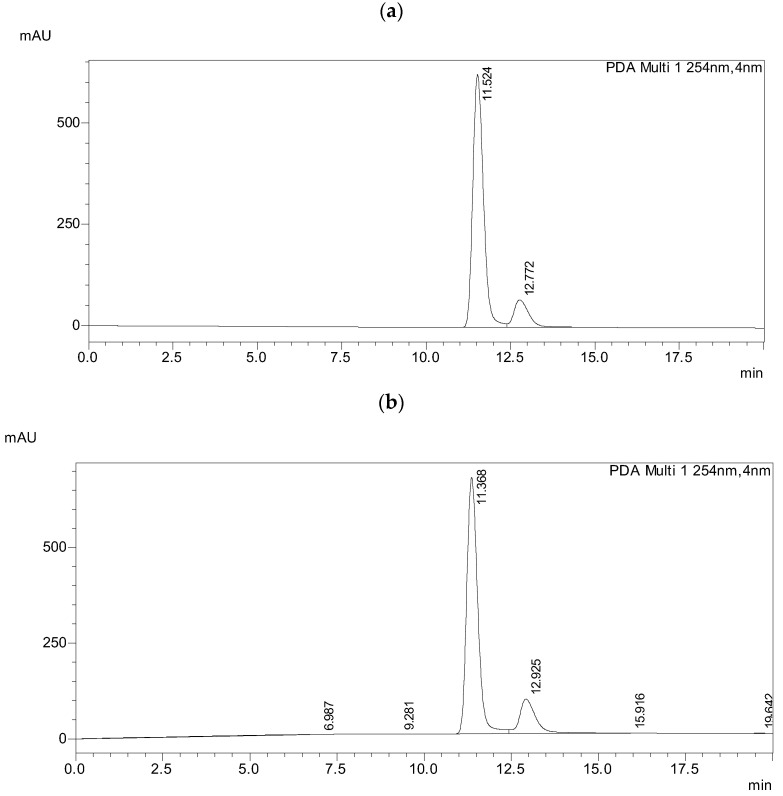
Chromatograms of (**a**) racemate 3PhPh (R,S) and (**b**) racemate 7PhPh (R,S) in the solvent system ACN/WATER in the ratio 99:1 (*v*/*v*) on the ReproSil Chiral-JM column, flow rate 0.3 mL/min.

On the ReproSil Chiral-JM column, baseline separation of both racemates was achieved in a short time with a resolution above 1.5, as shown in Figure 10a,b.

**Figure 11:**
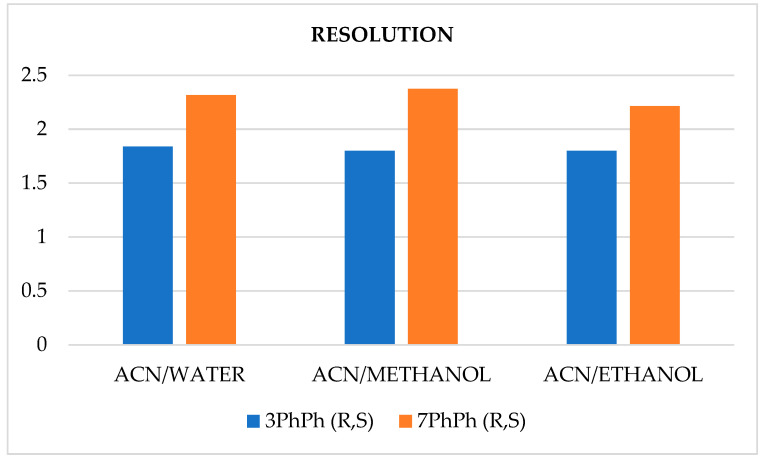
Resolution of racemates from different solvent systems on the ReproSil Chiral-JM column at a flow rate of 0.3 mL/min.

Enantioseparations were also tested on this column using ethanol and methanol in place of water, and the resolution results are illustrated in Figure 11.

Higher resolution was achieved in each case for the racemate with the longer oligomethylene spacer. The differences in resolution for the individual racemates were not significant, even when the solvent system was changed. The chromatographic parameters obtained on the ReproSil Chiral-JM column are summarized in Table 3. No significant differences in retention times or other parameters were observed when water was replaced with alcohol.

The four-ring racemic mixtures are best separated on a cellulose-packed column due to stronger interactions between the analytes and the stationary phase. It was found that the (S)-enantiomer elutes first in all cases. Interestingly, the three-ring liquid crystalline racemic esters with similar chemical structures did not separate on the ReproSil Chiral-JM column, regardless of the mobile phase. Here, the additional benzene ring enhances these interactions, facilitating baseline separation. Racemic esters with three benzene rings separated significantly better on amylose columns [15,18,19,20].

## 4. Conclusions

This study aimed to synthesize and characterize four-ring racemic smectics. Additionally, the goal was to separate these racemates into enantiomers using chiral liquid chromatography. Both racemates were synthesized with high purity. Dielectric studies confirmed the presence of two smectic phases in these racemates: the anticlinic phase (SmC_A_) and the smectic A phase (SmA). The racemates exhibit the anticlinic phase over a very wide temperature range, making them good admixtures for antiferroelectric liquid–crystalline mixtures [4,6,7,8,9,10,11,12,13,14,36,37,38,39]. Interestingly, the (S) enantiomers with the same structure also exhibit the synclinic smectic phase, SmC* (a ferroelectric phase) [40,41,42,43,44], which is not observed in the racemates. It seems to be a general rule that racemization, driven by molecules with opposite chirality, favors the formation of the anticlinic SmC_A_ phase rather than the synclinic SmC phase [18].

HPLC analyses revealed optimal conditions that enabled the baseline separation of both racemic mixtures into their enantiomers, with a resolution exceeding 1.5. The cellulose column proved to provide better results than the amylose column, undoubtedly due to the additional benzene ring, which enhances interactions with the chiral selector. We hope this procedure can be scaled up to preparative levels, enabling enantiomer separation without the need for lengthy synthesis.

Further chromatographic analyses are underway, but it is already clear that suitable enantioseparation conditions for the studied racemates can be found.

## Figures and Tables

**Table 1:** The structures and names of the chiral selectors (R = means amylose or cellulose) [19,34,35].

ReproSil Chiral-MIG **Amylose** tris(3-chloro-5-methylphenylcarbamate) 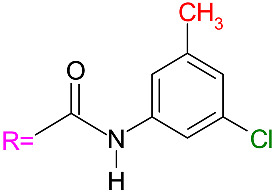	ReproSil Chiral-JM **Cellulose** tris(4-methylbenzoate) 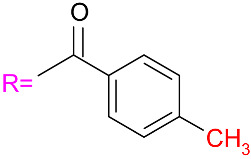

**Table 2:** Phase transition temperatures and enthalpies for the racemates.

Acronym	Phase Transition Behavior ^a^						
**3PhPh (R,S)**	**Cr**	68.9–71.3 °C 115.0 °C 77.4 °C *23.3 kJ/mol*	**SmC_A_**	192.2–194.3 °C 196.7 °C 195.7 °C *1.31 kJ/mol*	**SmA**	214.2–217.0 °C 212.2 °C 211.4 °C *2.07 kJ/mol*	**Iso**
**7PhPh (R,S)**	**Cr**	91.1–93.4 °C 89.5 °C 49.9 °C *21.9 kJ/mol*	**SmC_A_**	192.2–193.6 °C 188.2 °C 187.0 °C *0.84 kJ/mol*	**SmA**	214.8–217.3 °C 210.6 °C 211.0 °C *2.09 kJ/mol*	**Iso**

^a^ The first row contains POM measurements in the cooling cycle; the second row contains DSC measurements in the heating cycle; the third row contains DSC measurements in the cooling cycle; the fourth row contains enthalpies in the heating cycle.

**Table 3:** Chromatographic data—retention times, enantiomer elution order, selectivity, and number of theoretical plates (ReproSil Chiral-JM column).

Racemic Mixture	Mobile Phase (99:1) (*v*/*v*), 0.3 mL/min	t_r1_; t_r2_	EEO	α	N_S_; N_R_
**3PhPh (R,S)**	ACN/WATER ACN/METHANOL ACN/ETHANOL	11.524; 12.772 11.413; 12.637 11.397; 12.587	S ˃ R	1.186 1.185 1.182	6550; 4200 6330; 4120 6420; 4450
**7PhPh (R,S)**	ACN/WATER ACN/METHANOL ACN/ETHANOL	11.368; 12.925 11.359; 12.942 11.713; 13.154	S ˃ R	1.356 1.168 1.123	6480; 4380 6300; 4615 6990; 5030

## Data Availability

The original contributions presented in this study are included in the article/Supplementary Materials. Further inquiries can be directed to the corresponding author.

## Supplementary Materials

The following supporting information can be downloaded at https://www.mdpi.com/article/10.3390/ma19091719/s1, Figure S1: Mass spectrum for the racemate 3PhPh (R,S); Figure S2: Mass spectrum for the racemate 7PhPh (R,S); Figure S3: ^1^H NMR spectrum of the racemate 3PhPh (R,S) in CDCl_3_; Figure S4: ^13^C NMR spectrum of the racemate 3PhPh (R,S) in CDCl_3_; Figure S5: ^1^H NMR spectrum of the racemate 7PhPh (R,S) in CDCl_3_; Figure S6: ^13^C NMR spectrum of the racemate 7PhPh (R,S) in CDCl_3_; Figure S7: Real part of permittivity $\varepsilon^{’}$ versus temperature T for the racemate 3PhPh (R,S) (at cooling, no DC field) for several frequencies f of measuring signal (cell with gold electrodes, 5 μm thick, planar alignment). Two vertical scales are used to illustrate the phase transition of Iso-SmA; Figure S8: Real (a) and imaginary (b) parts of permittivity versus frequency f for the racemate 3PhPh (R,S) (at cooling, no DC field) for several temperatures T (cell with gold electrodes, 5 μm thick, planar alignment); Figure S9: Real part of permittivity $\varepsilon^{’}$ versus temperature T for the racemate 3PhPh (R,S) (at cooling, 5V DC field) for several frequencies f of measuring signal (cell with gold electrodes, 5 μm thick, planar alignment). Two vertical scales are used to illustrate the phase transition of Iso-SmA; Figure S10: Real (a) and imaginary (b) parts of permittivity versus frequency f for the racemate 3PhPh (R,S) (at cooling, 5V DC field) for several temperatures T (cell with gold electrodes, 5 μm thick, planar alignment); Figure S11: Real part of permittivity $\varepsilon^{’}$ versus temperature T for the racemate 7PhPh (R,S) (at cooling, no DC field (a) and 5V DC field (b)) for several frequencies f of measuring signal (cell with gold electrodes, 5 μm thick, planar alignment); Figure S12: Real (a) and imaginary (b) parts of permittivity versus frequency f for the racemate7PhPh (R,S): (at cooling, no DC field) for several temperatures T (cell with gold electrodes, 5 μm thick, planar alignment); Figure S13: Real (a) and imaginary (b) parts of permittivity versus frequency f for the racemate 7PhPh (R,S) (at cooling, 5V DC field) for several temperatures T (cell with gold electrodes, 5 μm thick, planar alignment); Figure S14: DSC curves for the racemate 3PhPh (R,S) (red in the heating cycle and blue in the cooling cycle); Figure S15: DSC curves for the racemate 7PhPh (R,S) (red in the heating cycle and blue in the cooling cycle); the phase transition at approximately 174 °C was not confirmed by polarizing microscopy and dielectric measurements; Figure S16: DSC curves for the enantiomer 3PhPh (S) (red in the heating cycle and blue in the cooling cycle); Figure S17: DSC curves for the enantiomer 7PhPh (S) (red in the heating cycle and blue in the cooling cycle). References [22,45,46,47] are cited in the Supplementary Materials.
