# Supplementary material for: Synthesis, Properties, and Enantioseparation of Four-Ring Racemic Smectics
Source: Materials (Basel). 2026 Apr 23;19(9):1719. doi: 10.3390/ma19091719 (PMC13164546; doi:10.3390/ma19091719)
Supplement: Supplementary file 1 [file materials-19-01719-s001.zip › materials-4247009-supplementary.pdf]

Synthesis, Properties, and Enantioseparation of Four-Ring Racemic Smectics

Edyta Wojda [1], Monika Zając [1], Paweł Perkowski [2] and Magdalena Urbańska [1,*]

[1] Institute of Chemistry, Military University of Technology, ul. Sylwestra Kaliskiego 2, 00-908 Warsaw, Poland; edyta.wojda@student.wat.edu.pl; monika.zajac@wat.edu.pl

[2] Institute of Applied Physics, Military University of Technology, ul. Sylwestra Kaliskiego 2, 00-908 Warsaw, Poland; pawel.perkowski@wat.edu.pl

* Correspondence: magdalena.urbanska@wat.edu.pl; +48-261-83-75-49

Mass spectra for the racemates

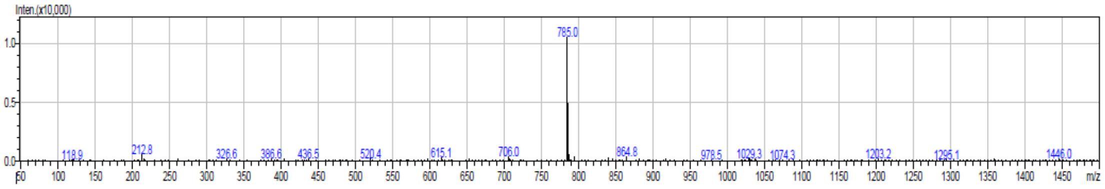

Figure S1. Mass spectrum for the racemate 3PhPh (R,S).

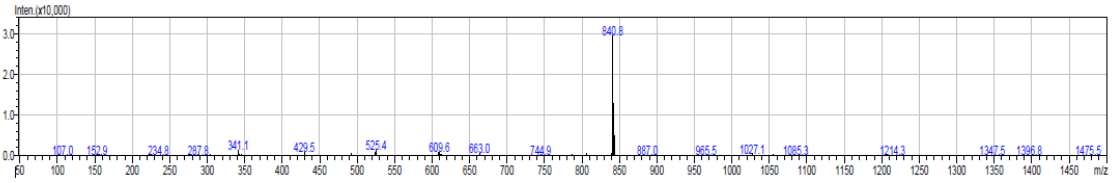

Figure S2. Mass spectrum for the racemate 7PhPh (R,S).

NMR spectra for the racemates

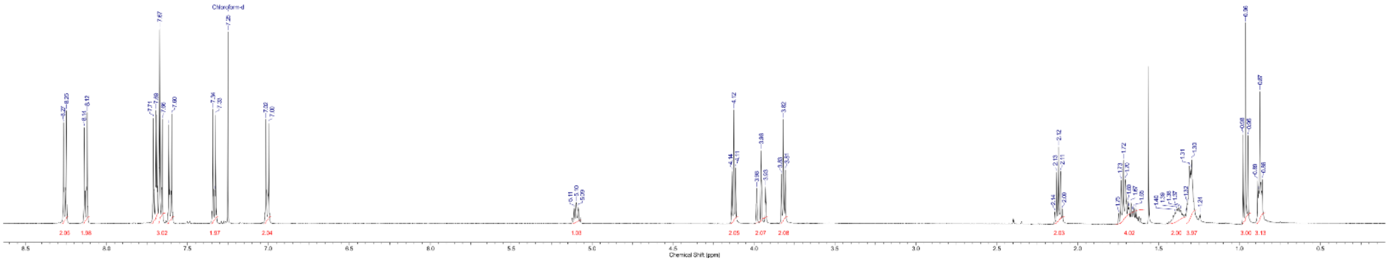

Figure S3. ^{1}H NMR spectrum of the racemate 3PhPh (R,S) in $CDCl_3$.

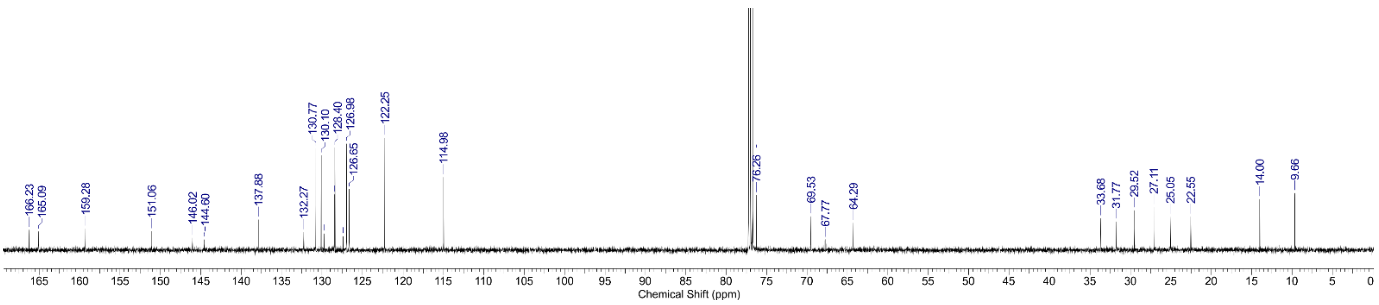

Figure S4. ^{13}C NMR spectrum of the racemate 3PhPh (R,S) in $CDCl_3$.

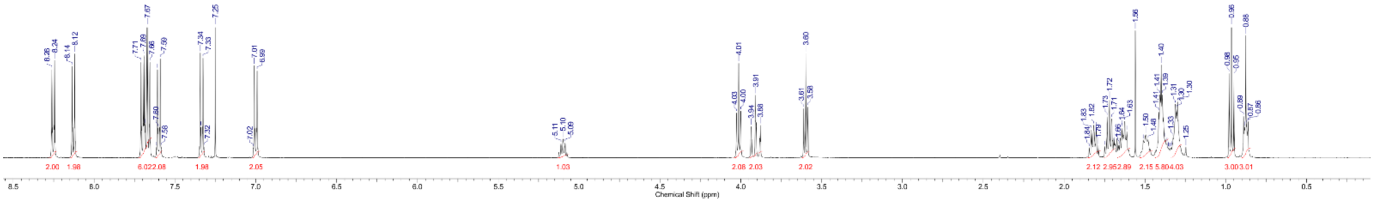

Figure S5. ^{1}H NMR spectrum of the racemate 7PhPh (R,S) in $CDCl_3$.

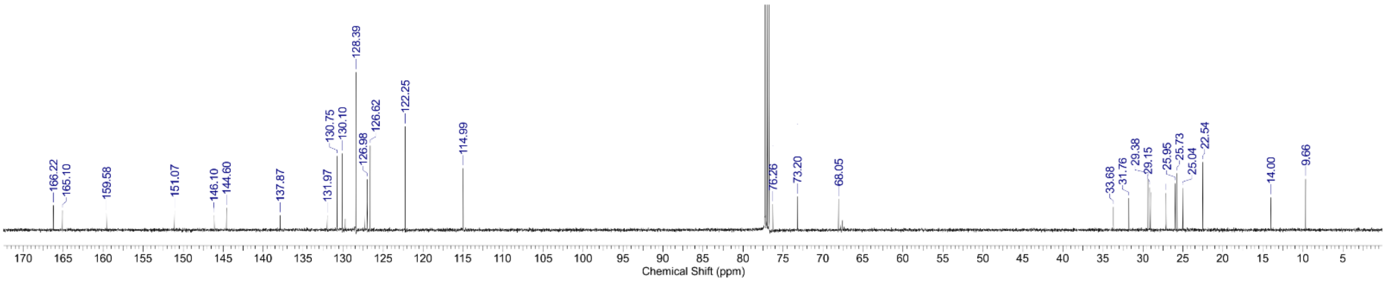

Figure S6. ^{13}C NMR spectrum of the racemate 7PhPh (R,S) in $CDCl_3$.

Dielectric measurements for the racemate 3PhPh (R,S)

Figure S7 presents the real part of the permittivity ε' versus temperature for the racemate with a shorter oligomethylene spacer (r = 3). One can observe that between an isotropic liquid and a molecular crystal, two phases are present: the SmA and SmC$_A$ phases. Orthogonal SmA phase appears at a temperature of 220 °C, while crystallization temperature is around 94 °C. Between 220 °C and 203 °C, one observes the racemic SmA phase without any significant dispersion. Below 203 °C, the racemic SmC$_A$ phase exhibits a rich dispersion.

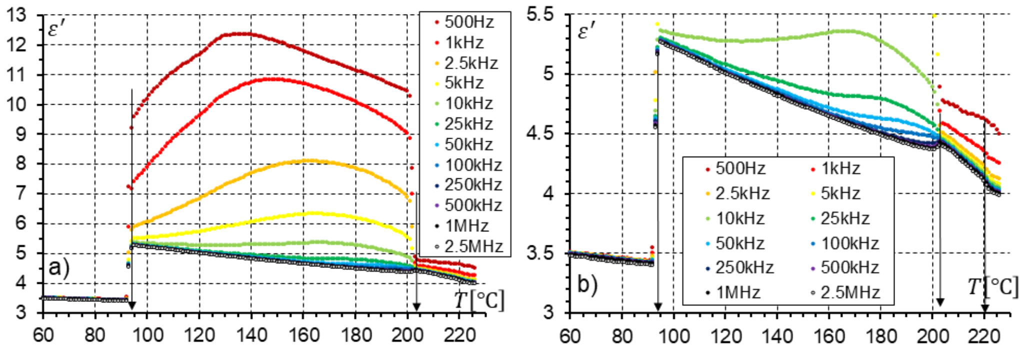

Figure S7. Real part of permittivity ε' versus temperature T for the racemate 3PhPh (R,S) (at cooling, no DC field) for several frequencies f of measuring signal (cell with gold electrodes, 5 μm thick, planar alignment). Two vertical scales are used to illustrate the phase transition of Iso-SmA.

To analyze dispersion in the racemic SmCA phase, two plots are presented: Figures S8(a) and S8(b). Figure S8(a) presents the real part of permittivity ε' versus frequency for several temperatures in the SmA and SmCA phases. In contrast, Figure S8(b) presents the imaginary part of permittivity ε'' versus frequency for the same temperatures as Figure S7(a). At 210 °C (the SmA phase), we do not see any dispersion (apart from ions' contribution at low frequency [45] and parasitic effect at high frequency [22]). In the SmCA phase, two relaxations are visible: one stronger relaxation with the relaxation at low (~1 kHz) frequency (this relaxation is well seen in the whole SmCA phase), and one weaker relaxation with the relaxation at high frequency (~100 kHz). This relaxation is observed only at high temperatures (200 °C) in the SmCA phase. The first relaxation can be interpreted as P_L-mode, while the second is molecular S-mode. The third mode (P_H) present in the enantiomeric SmCA* phase is not observed in the racemic SmCA phase.

Both modes should be described as fulfilling the Arrhenius law [46]. It seems that P_L-mode fulfils this law. In the case of S-mode, it is difficult to confirm it.

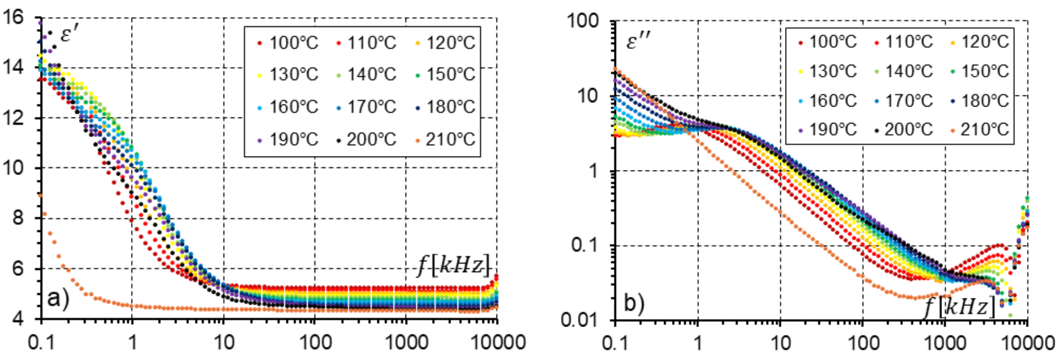

Figure S8. Real (a) and imaginary (b) parts of permittivity versus frequency f for the racemate 3PhPh (R,S) (at cooling, no DC field) for several temperatures T (cell with gold electrodes, 5 μm thick, planar alignment).

To confirm the origin of both modes, measurements were conducted using a 5 V DC field. The results are presented in Figures S9 and S10. Figures S9(a) and S9(b) show the real part of the permittivity ε' versus temperature. One can see that the electric response in measurements with a DC field is slightly stronger than in those without.

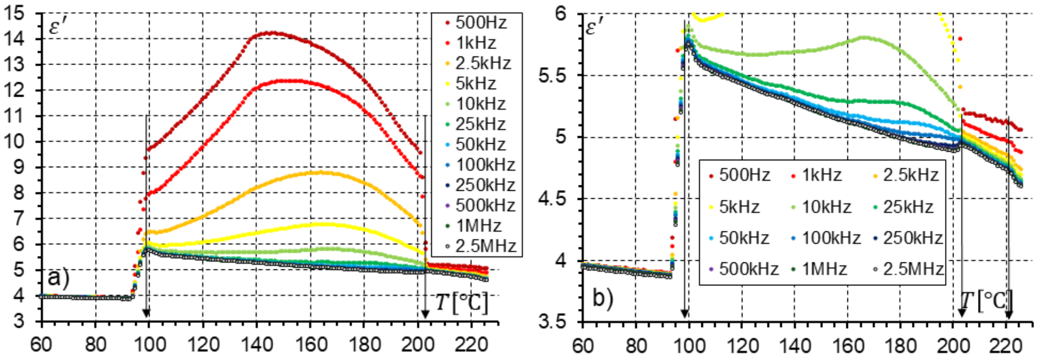

Figure S9. Real part of permittivity ε' versus temperature T for the racemate 3PhPh (R,S) (at cooling, 5V DC field) for several frequencies f of measuring signal (cell with gold electrodes, 5 μm thick, planar alignment). Two vertical scales are used to illustrate the phase transition of Iso-SmA.

Figures S10(a) and S10(b) show the real ε' and imaginary ε'' parts of permittivity. Both modes are strengthened under a DC field. Additionally, due to the reduction of ions' contribution, P_L-mode is better visible. Simultaneously, the molecular S-mode is now more visible. We see it for temperatures 200, 190, and 180 °C.

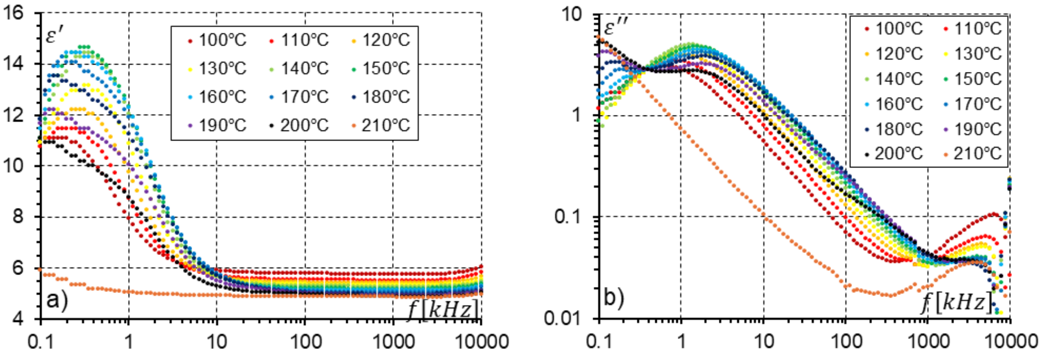

Figure S10. Real (a) and imaginary (b) parts of permittivity versus frequency f for the racemate 3PhPh (R,S) (at cooling, 5V DC field) for several temperatures T (cell with gold electrodes, 5 μm thick, planar alignment).

The questions arise: Why does S-mode exhibit a low relaxation frequency, despite involving individual molecular motions, which should be relatively fast? And the second: Why is the molecular S-mode visible in the racemate 3PhPh (R,S) at high temperatures, while it is not visible at low

temperatures? To answer these questions, we measured the racemate 7PhPh (R,S) and discussed the electric properties of both materials.

Dielectric measurements for the racemate 7PhPh (R,S)

Figure S11 presents the real part of permittivity ε' versus temperature for the racemate with a longer oligomethylene spacer (r = 7). Figure S11(a) shows the results without a DC field, while Figure S11(b) shows the results with a 5 V DC field. The isotropic liquid transforms into the SmA phase at 222 °C. This temperature is similar to the clearing temperature of the racemate 3PhPh (R,S). The racemic SmA phase transforms into the racemic SmC_A at 196 °C. This temperature is 12 degrees lower than the SmA-SmC_A phase transition in the racemate 3PhPh (R,S). The DC field enhances the electric response compared to those without a DC field. The racemic SmC_A phase exhibits one relaxation. It is detectable at temperatures ranging from 196 °C to 100 °C. This relaxation can be interpreted as a molecular S-mode. It is better visible in the electric response than S-mode in the racemate 3PhPh (R,S) because the collective P_L-mode is invisible in the racemate 7PhPh (R,S). Relatively strong collective P_L-mode in the racemate 3PhPh (R,S) makes S-mode undetectable for lower temperatures. The question arises: Why do we not see P_L-mode in the electronic response of the racemate 7PhPh (R,S)? To answer this question, we should analyze the structure of both molecules. Both 3PhPh (R,S) and 7PhPh (R,S) molecules are heavy because they have four benzene rings. Such molecules are heavy components of dual-frequency nematic mixtures [47]. Their relaxation frequency of S-mode is low (it defines the crossover frequency in dual nematics). The racemate 7PhPh (R,S) is heavier and longer than the racemate 3PhPh (R,S); hence, S-mode in the racemate 7PhPh (R,S) is slower than in the racemate 3PhPh (R,S). Additionally, P_L-mode in the racemate 7PhPh (R,S) should be slower than P_L-mode in the racemate 3PhPh (R,S). At first glance, one might think that P_L-mode probably does not exist in the racemate 7PhPh (R,S). However, it appears to exist, but its relaxation frequency is too low to be detectable within the current measurement range. Additionally, Figure S11 shows that S-mode in the racemate 7PhPh (R,S) fulfils the Arrhenius law.

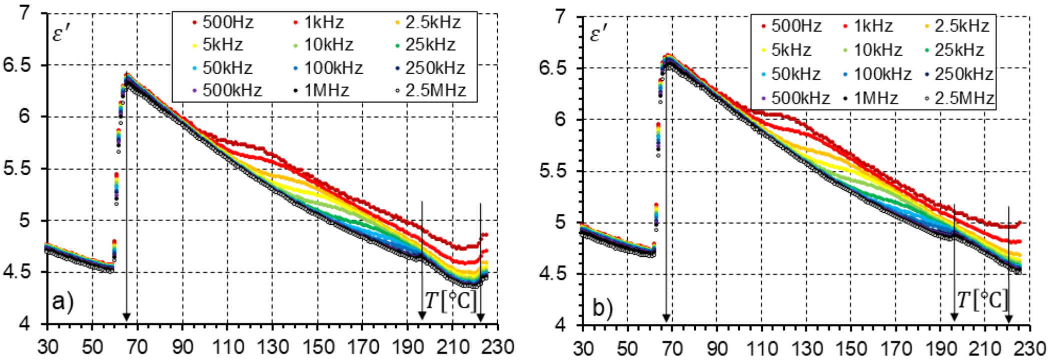

Figure S11. Real part of permittivity ε' versus temperature T for the racemate 7PhPh (R,S) (at cooling, no DC field (a) and 5V DC field (b)) for several frequencies f of measuring signal (cell with gold electrodes, 5 μm thick, planar alignment).

To analyze dispersion in the racemic SmC_A phase, two plots are presented: Figures S12(a) and S12(b). Figure S12(a) presents the real part of permittivity ε' versus frequency for several temperatures in the SmA and SmC_A phases. In contrast, Figure S12(b) presents the imaginary part of permittivity ε'' versus frequency. At 210 °C and 200 °C (SmA phase), we do not see any dispersion (apart from ions' contribution at low frequency [45] and parasitic effect at high frequency [22]). In the SmC_A phase, one weak relaxation is visible. Its relaxation frequency changes from 100 kHz at high temperature (190 °C) to less than 1 kHz at low temperature(100 °C). This mode fulfills the Arrhenius law [46].

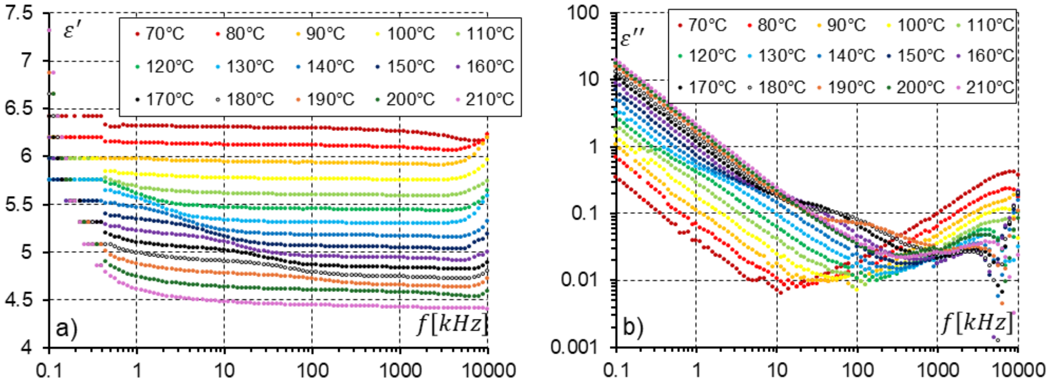

Figure S12. Real (a) and imaginary (b) parts of permittivity versus frequency f for the racemate7PhPh (R,S): (at cooling, no DC field) for several temperatures T (cell with gold electrodes, 5 μm thick, planar alignment).

The results obtained with a 5 V DC field are presented in Figure S13. Figure S13(a) presents the real part of permittivity ε' versus frequency for several temperatures in the SmA and SmC_A phases. In contrast, Figure S13(b) presents the imaginary part of permittivity ε'' versus frequency. The DC field slightly raises the real part of permittivity while suppressing the ion contribution to the imaginary part of permittivity. Hence, S-mode is better visible after the movement of DC-suppressing ions.

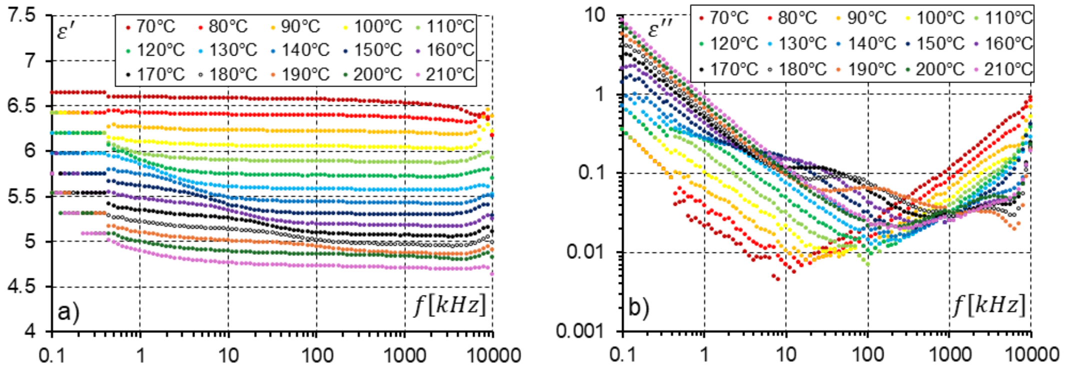

Figure S13. Real (a) and imaginary (b) parts of permittivity versus frequency f for the racemate 7PhPh (R,S) (at cooling, 5V DC field) for several temperatures T (cell with gold electrodes, 5 μm thick, planar alignment).

DSC curves for the racemates and enantiomers

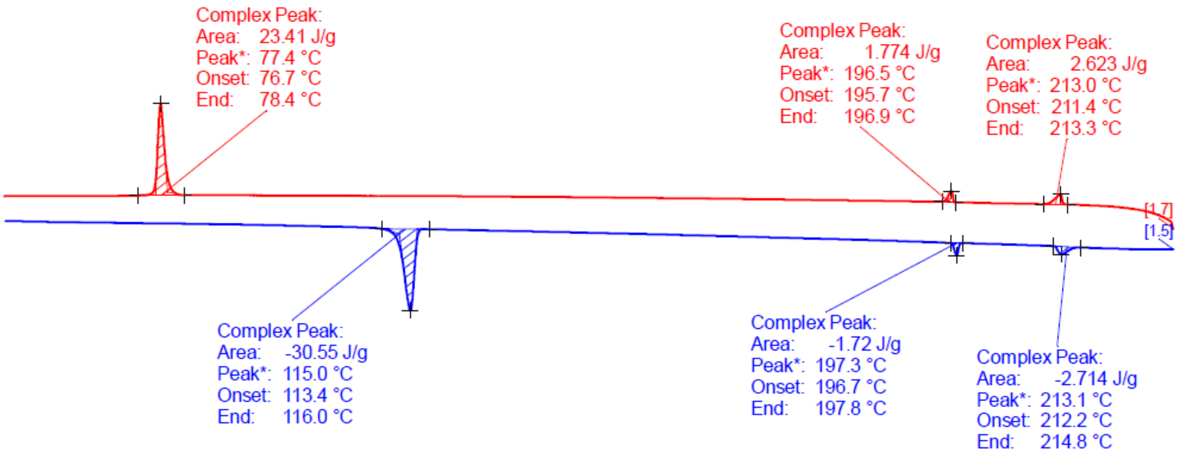

Figure S14. DSC curves for the racemate 3PhPh (R,S) (red in the heating cycle and blue in the cooling cycle).

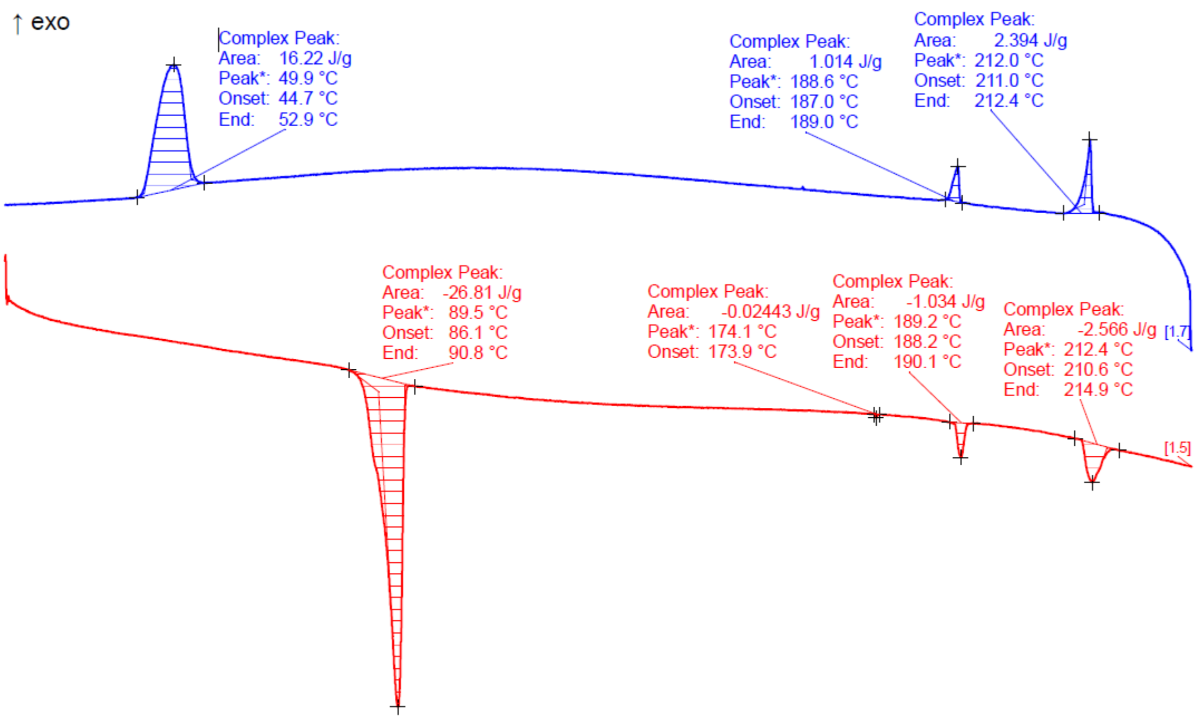

Figure S15. DSC curves for the racemate 7PhPh (R,S) (red in the heating cycle and blue in the cooling cycle); the phase transition at approximately 174°C was not confirmed by polarizing microscopy and dielectric measurements.

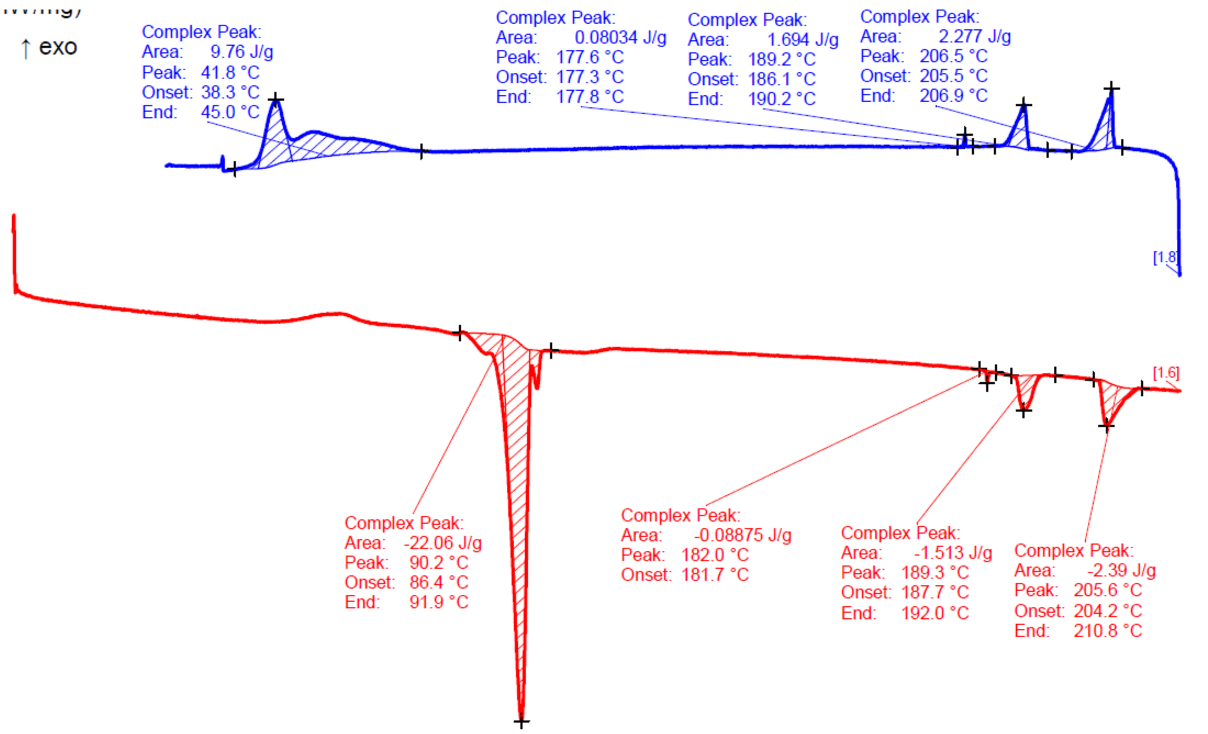

Figure S16. DSC curves for the enantiomer 3PhPh (S) (red in the heating cycle and blue in the cooling cycle).

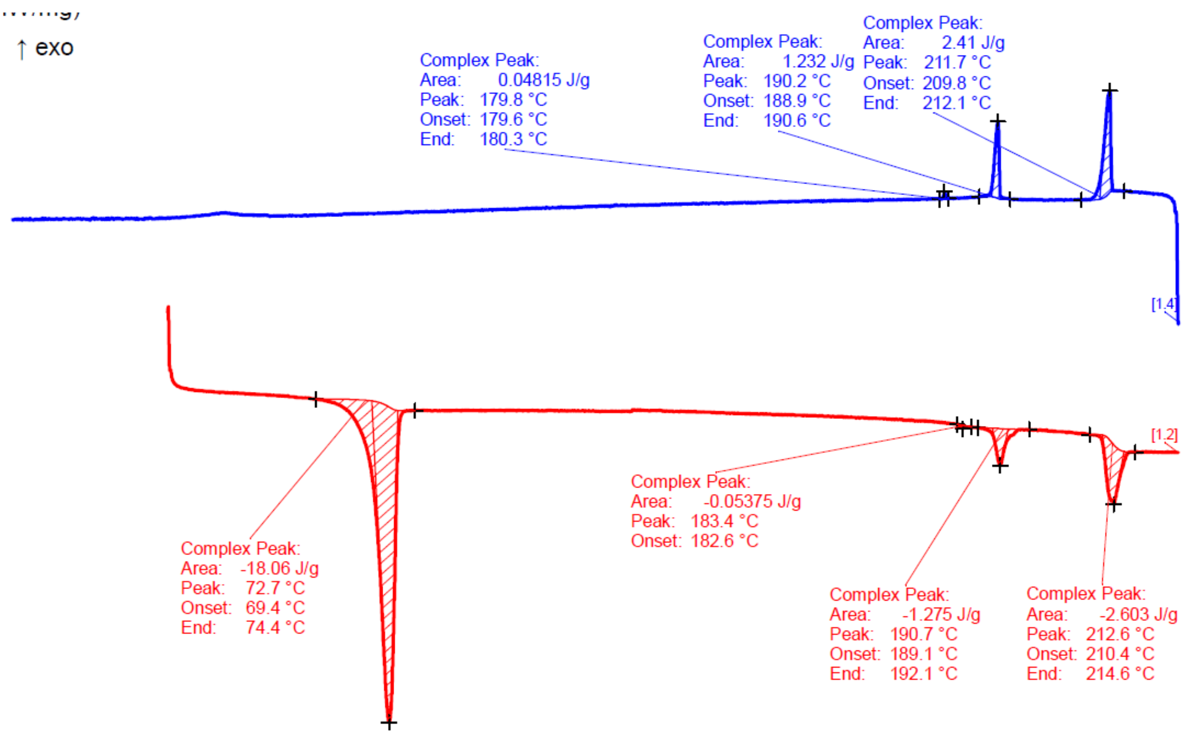

Figure S17. DSC curves for the enantiomer 7PhPh (S) (red in the heating cycle and blue in the cooling cycle).
